# Light-Induced Sulfur Transport inside Single-Walled Carbon Nanotubes

**DOI:** 10.3390/nano10050818

**Published:** 2020-04-25

**Authors:** Olga V. Sedelnikova, Olga A. Gurova, Anna A. Makarova, Anastasiya D. Fedorenko, Anton D. Nikolenko, Pavel E. Plyusnin, Raul Arenal, Lyubov G. Bulusheva, Alexander V. Okotrub

**Affiliations:** 1 Nikolaev Institute of Inorganic Chemistry SB RAS, 630090 Novosibirsk, Russia; gurova@niic.nsc.ru (O.A.G.); fedorenko@niic.nsc.ru (A.D.F.); plus@niic.nsc.ru (P.E.P.); spectrum@niic.nsc.ru (A.V.O.); 2 Physical Chemistry, Institute of Chemistry and Biochemistry, Free University of Berlin, 14195 Berlin, Germany; anna.makarova@fu-berlin.de; 3 Budker Institute of Nuclear Physics SB RAS, 630090 Novosibirsk, Russia; a.d.nikolenko@inp.nsk.su; 4 Laboratorio de Microscopias Avanzadas, Instituto de Nanociencia de Aragón, Universidad de Zaragoza, 50018 Zaragoza, Spain; arenal@unizar.es; 5 Research & Development Agency of Aragon ARAID Foundation, 50018 Zaragoza, Spain; 6 Instituto de Ciencias de Materiales de Aragon, CSIC-U. de Zaragoza, 50009 Zaragoza, Spain; 7 Laboratory of Carbon Nanomaterials, Novosibirsk State University, 630090 Novosibirsk, Russia

**Keywords:** single-walled carbon nanotubes, sulfur, encapsulation, de-encapsulation, illumination, X-Ray spectroscopy

## Abstract

Filling of single-walled carbon nanotubes (SWCNTs) and extraction of the encapsulated species from their cavities are perspective treatments for tuning the functional properties of SWCNT-based materials. Here, we have investigated sulfur-modified SWCNTs synthesized by the ampoule method. The morphology and chemical states of carbon and sulfur were analyzed by transmission electron microscopy, Raman scattering, thermogravimetric analysis, X-ray photoelectron and near-edge X-ray absorption fine structure spectroscopies. Successful encapsulation of sulfur inside SWCNTs cavities was demonstrated. The peculiarities of interactions of SWCNTs with encapsulated and external sulfur species were analyzed in details. In particular, the donor–acceptor interaction between encapsulated sulfur and host SWCNT is experimentally demonstrated. The sulfur-filled SWCNTs were continuously irradiated *in situ* with polychromatic photon beam of high intensity. Comparison of X-ray spectra of the samples before and after the treatment revealed sulfur transport from the interior to the surface of SWCNTs bundles, in particular extraction of sulfur from the SWCNT cavity. These results show that the moderate heating of filled nanotubes could be used to de-encapsulate the guest species tuning the local composition, and hence, the functional properties of SWCNT-based materials.

## 1. Introduction

Carbon nanotubes (CNTs) are an ideal material for filling of their empty cavities with different species. Almost any compound can be encapsulated inside CNT through liquid-phase capillarity and vapour-phase diffusion effects or by *in situ* filling during CNT growth [1,2,3,4,5]. The quasi-one dimensional structure of CNTs templates the synthesis of metallic nanowires [6,7,8], ordered nanoparticles [9,10] and even ordered atomic chains [10]. The obtained hybrid materials usually exhibit novel functional properties with respect to the empty nanotubes and bulk guest species mainly due to atypical low-dimensional structure and specific interaction between the components, such as magnetic, donor–acceptor, etc. [4,11,12,13,14,15,16,17,18,19].

The reverse process, i.e., exclusion of the guest material from the CNT cavity, is also important because partial cleaning of the nanotubes’ cavities can be useful for local tuning of their properties. The enhancement of optoelectronic properties of single-walled carbon nanotubes (SWCNTs) through controlled encapsulation and de-encapsulation of guest molecules from their cavities by choosing of an appropriate solvent was discussed in Ref [11]. Another approach is associated with the impact of high-energy irradiations on nanotubes. For example, electron beam irradiation of CNTs filled with CuI caused removal of iodine and formation of a copper core, which should endow the material with different functionalities [20]. The other example is drug release from encapsulating CNTs to target the cancer cell under heating [21,22,23].

Here, we report the de-encapsulation of the guest species from the cavities of SWCNTs under synchrotron illumination of sulfur-filled SWCNTs. The choice of sulfur as the guest was dictated by a high efficiency of SWCNTs filling and a low melting point of sulfur. The work is based on the comparison of transmission electron microscopy (TEM) images, thermogravimetric (TG) data and Raman spectra of SWCNTs containing encapsulated and coated sulfur, as well as X-ray photoelectron spectra (XPS) and near-edge X-ray absorption fine structure (NEXAFS) spectra of the samples before and after continuous *in situ* illumination with polychromatic photon beam of high intensity (zero-order light from the dipole beamline of BESSY II synchrotron radiation facility). Although sulfur-filled nanotubes have been investigated earlier [17,18,24,25,26,27,28], we, for the first time, systematically compare the stability and chemical state of encapsulated sulfur atoms with those composing the sulfur particles decorated the external surface of SWCNTs. Moreover, our results provide the experimental proof of the donor–acceptor interaction between encapsulated sulfur and host SWCNT that was suggested previously [17,18,28].

## 2. Materials and Methods 

### 2.1. Materials Preparation

SWCNTs (OCSiAl company, Novosibirsk, Russia, LOT # 50-04122014) with a diameter range of about 1.7–1.9 nm were used in this work. The raw nanotubes are entangled bundles with an average diameter of *ca.* 100–200 nm [29]. We purified SWCNTs using the three-step procedure described elsewhere [29]. It included (1) heating in air at 500 °C for 30 min, (2) treatment by HCl to wash the accessible residual iron catalyst, and (3) magnetic treatment. The first step removes amorphous carbon and opens tips of the CNTs. HCl acid dissolves both the external and the endohedral catalyst particles reducing the iron content to *ca.* 1.8 wt% [29]. Note that the obtained purity is superior in comparison with the results obtained for OCSiAl SWCNTs treated merely by HCl without pre-heating step [30]. The following magnetic treatment allows extracting nanotubes containing the majority of residual endohedral catalyst particles. After three-step purification procedure, the iron content in SWCNTs sample was *ca.* 0.3 wt% [29]. This sample labeled further as “initial SWCNTs” is used as a reference sample.

One portion of the acid-purified nanotubes was filled with sulfur. Briefly, 10mg SWCNTs (repeatedly tip-opened by heating to 500 °C in air for 30 min) and 40 mg sulfur were grinded and placed in a quartz ampoule. The ampoule was pumped out to 0.01 atm, sealed and heated in a furnace at 750 °C for 1 h followed by heating at 600 °C for 14 h (heating rate 400 °C/h). Finally, the ampoule was naturally cooled to room temperature. The obtained product was marked as SWCNT/S-1. Another portion of acid-purified SWCNTs was filtered in magnetic field and used for ampoule filling with sulfur following the method described above. After sonication of the second product in toluene for 10 min using an ultrasound bath, we obtained the sample marked as SWCNT/S-2.

### 2.2. Characterization Methods

The structure of materials was characterized by TEM on a JEOL 2010 microscope (JEOL Ltd., Tokyo, Japan). High-angle annular dark field scanning TEM (HAADF-STEM) imaging studies were performed on probe-corrected FEI Titan Low-Base 60-300 microscope (FEI-Thermo Fisher Scientific, Eindhoven, Netherland) operating at 80 kV (fitted with a X-FEG® gun, a Cs-probe corrector (CESCOR from CEOS GmbH)). In addition, high-resolution TEM (HRTEM) imaging studies were carried out in a FEI Titan Cube 60–300 microscope (FEI-Thermo Fisher Scientific, Eindhoven, Netherland) operating at 80 kV.

Raman scattering spectra were recorded at room temperature on a LabRAM HR Evolution spectrometer (Horiba, Kyoto, Japan) using a 514 nm excitation. The spectra were acquired at 3–5 points and averaged. TG analysis (TGA) was carried out with a Netzsch STA 449 F1 Jupiter1 thermal analyzer (Selb/Bayern, Germany). Samples (2–4 mg) were heated at a rate of 10 °C min^–1^ in an argon flow (10 ml/min) within temperature range of 30–1000 °C.

XPS and C *K*-edge NEXAFS spectra were recorded at the Berlin Elektronenspeicher ring für Synchrotronstrahlung (BESSY II, Helmholtz-Zentrum Berlin, Berlin, Germany) using radiation from the Russian-German beamline (RGBL). A powder sample was applied to a scratched copper substrate and placed into a chamber with base pressure of 10^-9^ mbar. The C *K*-edge NEXAFS spectra were recorded in the total-electron yield mode with an experimental resolution better than 0.1 eV. XPS spectra were measured using a monochromatic radiation with a photon energy of 400 eV. Overall XPS spectra measured using PHOIBOS 150 spectrometer (SPECS Surface Nano Analysis GmbH, Berlin, Germany) were used to determine the relative ratio of carbon and sulfur on the sample surface. After subtraction of a Shirley background, the C 1*s* and S 2*p* spectra were fitted using Gaussian/Lorentzian functions within Casa XPS 2.3.15 software (Casa Software Ltd., Teignmouth, UK). Energies of XPS spectral lines were calibrated to the Au 4f_7__/__2_ component position with an accuracy of ca. 0.05 eV. 

The S *K*-edge NEXAFS spectra were measured in the transmission mode using a precision silicon photodiode SPD [31] at beamline Cosmos at the VEPP-4M storage ring of BINP SB RAS [32,33]. The ring current was about 5-10 mA at 1.9 GeV. Si(111) double crystal monochromator with an energy resolution (Δ*E*/*E*) of 1 × 10^−4^ was used. The spectra were calibrated to the maximum of white line of sulfur powder at 2472.4 eV and CaSO_4_ at 2482.6 eV.

SWCNT/S-1 and SWCNT/S-2 were irradiated *in situ* with zero-order light from the Russian-German dipole Beamline of BESSY II [34,35,36]. Such radiation ranges from visible light to soft X-rays. The total flux of polychromatic photons was about 10^14^ photons/s. The scheme of our experiment included three steps: (1) measurement of XPS and NEXAFS spectra of a sample; (2) illumination of a sample by zero-order light for 30 s; (3) repeated measurement of the spectra. We did not move a samples during whole experiment, so, we are sure that we measured the same area of the sample before and after the treatment, i.e., the observed changes of the sulfur content does not related to inhomogeneity of our sample. The irradiated samples are marked as SWCNT/S-1i and SWCNT/S-2i.

## 3. Results and Discussion

### 3.1. Ampoule Filling of SWCNTs with Sulfur

**Figure 1:**
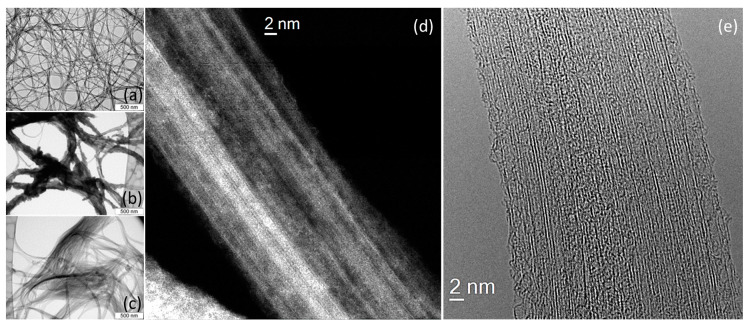
TEM images of initial SWCNTs (**a**), SWCNT/S-1 (**b**) and SWCNT/S-2 (**c**). HAADF-STEM (**d**) and HRTEM (**e**) images of a bundle of SWCNT/S-2.

The purification procedure results in partial de-bundling of nanotubes, the metallic impurities are not revealed (Figure 1a). Typical TEM images of SWCNTs with sulfur are presented in Figure 1b,c. SWCNT/S-1 sample contained the abundance of sulfur particles deposited on the outer side of nanotube bundles bonding them together toughly. After the toluene treatment, SWCNT bundles became more sparse due to removal of the excessive sulfur. Figure 1d,e present the HAADF-STEM and HRTEM images of SWCNT/S-2 sample, respectively, clearly showing nanotubes’ walls and sulfur inside their cavities [37,38,39] thus indicating that sulfur penetrates inside nanotubes and stays there after the washing of the sample with toluene. For filled nanotubes in a bundle, sulfur stuffs the cavity homogeneously.

**Figure 2:**
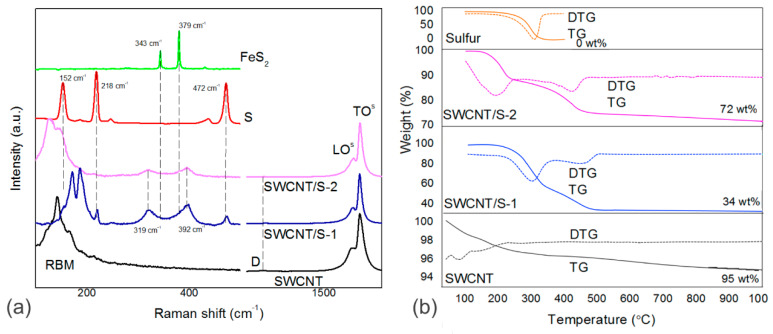
Raman spectra (**a**) and thermal analysis curves (**b**) of initial SWCNT, SWCNT/S-1 and SWCNT/S-2 samples in comparison with reference spectra of sulfur and pyrite.

Raman spectra of the samples are presented in Figure 2a exhibiting two regions related to the radial breathing modes (RBMs) and vibration of sulfur species (100–700 cm^−1^) and the first-order region (1250–1800 cm^−1^) for vibrations of carbon hexagons. A negligible intensity of the disorder-induced D-band in the spectrum of initial SWCNTs indicates a low defect density in the sample. The addition of sulfur changed the low-wavenumber spectra significantly, while the crystallinity of nanotubes was kept. The RBM modes of SWCNT/S-1 are upshifted as compared to those for the initial SWCNTs that may be related to the effect of external pressure from sulfur deposited on the side of nanotube ropes and the related strain affected on the radial vibrations of nanotubes. The spectrum of SWCNT/S-2 sample containing mainly the encapsulated species exhibits the RBM modes shifted back to their initial positions that is in agreement with previous works [18,27]. The spectra of sulfur-modified SWCNTs showed new wide bands located at *ca.* 319 can 392 cm^−1^, which were previously assigned to vibrations of encapsulated sulfur chains [18,27], however, the vibrations of pyrite also could contribute to these bands. Moreover, the spectra of SWCNT/S-1 sample exhibited rather intense bands of orthorhombic sulfur related to the external sulfur particles. The profile of the G-band is typical for semiconducting SWCNTs, due to the absence of a broad metallic component located at 1530–1545 cm^−1^ [40,41]. The observed narrow peak at 1593 cm^−1^ and small shoulder at 1575 cm^−1^ could be assigned to the longitudinal optical (LO) and transverse optical (TO) phonons in the semiconducting nanotubes [41]. The addition of sulfur has almost no effect on the position of G-band.

Figure 2b depicts the TG and differential TG (DTG) curves of the initial SWCNTs and the SWCNTs with sulfur. Heating of the initial SWCNTs showed a maximum weight loss at 40–220 °C (ca. 3 wt%), which was assigned to evaporation of adsorbed water molecules. The total weight loss at 1000 °C was about 5 wt%. High thermal stability of initial SWCNTs indicates good crystallinity of nanotubes’ wall that agrees the Raman data. The curves of elemental sulfur showed one evaporation process between 180 and 320 °C. SWCNT/S-1 sample was decomposed in two stages with a total weight loss of about 66 wt%. The material lost about 38 wt% in a temperature range of 200–320 °C due to removal of external sulfur [24]. The second weight loss of about 28 wt% observed between 320 and 500 °C corresponds to the evaporation of residual external sulfur and encapsulated sulfur. The absence of weight loss above 500 °C suggests the absence of a strong bonding between sulfur and carbon [24]. The TG curve of SWCNT/S-2 sample also revealed two stages at 100–250 °C (11 wt%) and 250–470 °C (13 wt%). The former step could be attributed to evaporation of sulfur encapsulated inside the outer SWCNTs, and, probably, evaporation of residual toluene molecules, which can be captured between the nanotubes. The latter step of weight loss is caused by the evaporation of sulfur encapsulated in the nanotubes located deeper inside a bundle that requires higher temperatures. The sulfur content in SWCNT/S-1 and SWCNT/S-2 samples could be determined as 66 and 24 wt%, respectively. The latter value is higher than the value reported for SWCNTs filled with sulfur by a similar method (*ca.* 12 wt% and 16 wt% from TG and XPS analysis [17]).

**Figure 3:**
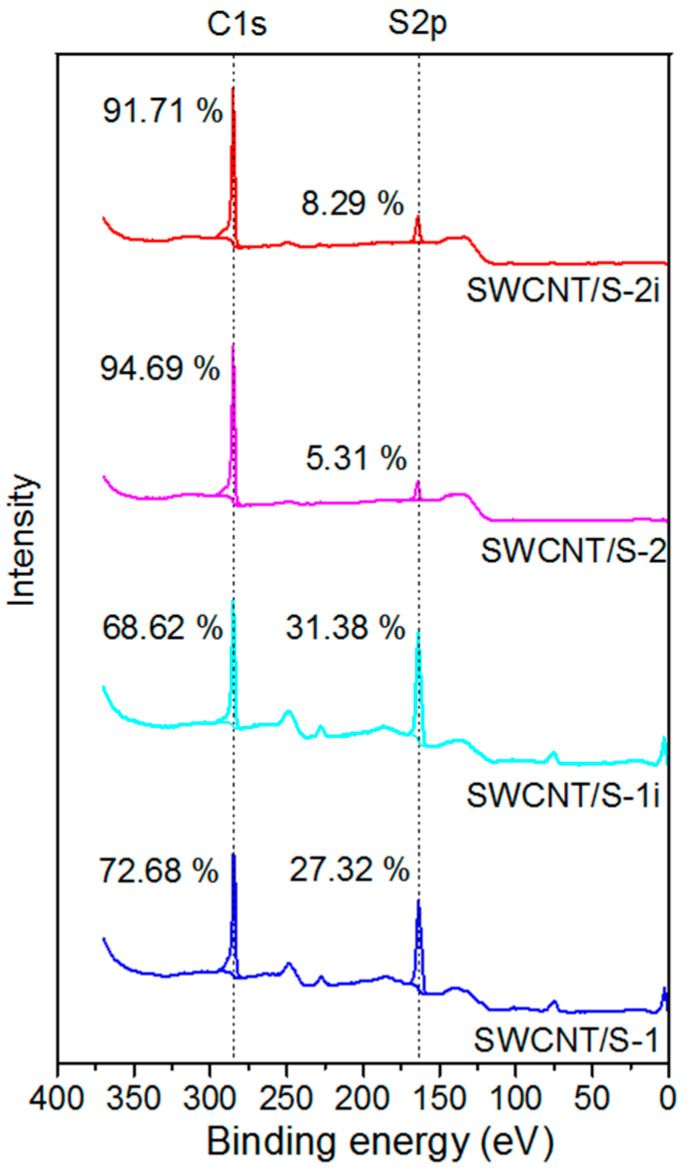
Overall XPS spectra of sulfur-contained SWCNTs before and after illumination.

According to the overall XPS, the relative proportion of carbon to sulfur (*n*_C_/*n*_S_) was about 72.7%/27.3% in SWCNT/S-1 (Figure 3). After the toluene treatment the sulfur content decreased to 5.3% (*n*_C_/*n*_S_ = 94.7%/5.3%). Our previous XPS study detected about 2.7 at% of oxygen and about 1.8 at% of iron in the HCl-purified SWCNTs and about 0.3 at% of iron after the magnetic treatment of SWCNTs [29]. Taking into account that the ampoules were pumped out, one can expect that the surfaces of SWCNT/S-1 and SWCNT/S-2 samples mainly contain carbon and sulfur.

**Figure 4:**
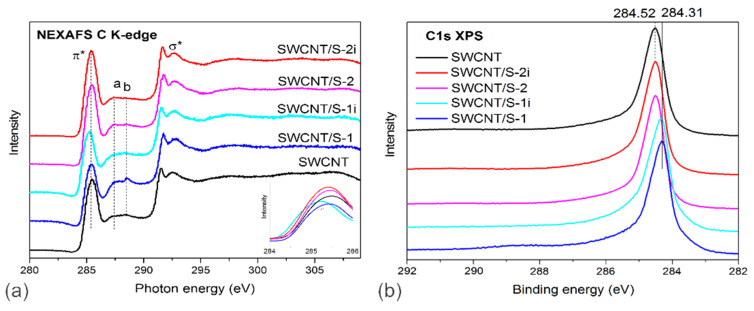
C *K*-edge NEXAFS (**a**) and C 1*s* XPS (**b**) spectra of initial SWCNTs and sulfur-contained SWCNTs before and after illumination. The insert in (**a**) compares π* resonances of the spectra normalized to the intensity of σ* resonances.

The chemical state of carbon in all samples were compared using C *K*-edge NEXAFS and C 1*s* XPS spectra (Figure 4). C *K*-edge spectra of initial and sulfur-modified SWCNTs showed two sharp peaks assigned to 1*s*→π* and 1*s*→σ* transitions (denoted as π* and σ* in Figure 4a). The sharpness of π* and σ* resonances and narrowness of C 1*s* line (Figure 4b) evidenced a good ordering of carbon atoms within SWCNT walls in agreement with the Raman and TG data. Moreover, the spectra exhibited weak peaks at 287.4 and 288.5 eV labelled *a* and *b*, which are likely contributed by carbon bounded with sulfur, oxygen and hydrogen [42,43,44]. Comparison of the spectra of initial SWCNTs and SWCNT/S-1 sample showed a decrease of the intensity of π* peak and an increase of intensity of the peaks *a* and *b* after addition of external sulfur particles. The following removal of the excessive sulfur enhanced the π* resonance and decreased the intensity of the peaks *a* and *b* with respect to the initial SWCNTs indicating effective washing of SWCNTs’ walls from contaminations [44]. The C 1*s* XPS spectra of the initial SWCNTs, SWCNT/S-1 and SWCNT/S-2 exhibited an asymmetric line (Figure 4b), corresponding to the *sp*^2^-hybridized carbon. For SWCNT/S-1 sample, C 1*s* line is downshifted by ca. 0.2 eV. The similar shift has been previously reported for CNTs coated by MoS_2_ [45] and SWCNTs filled with Hg_2_Cl_2_ [14] and AgCl [40] and related to the decrease in the Fermi level energy due to the *p*-doping of nanotubes. The removal of sulfur coating returns C 1*s* line back to the value for pristine SWCNTs.

**Figure 5:**
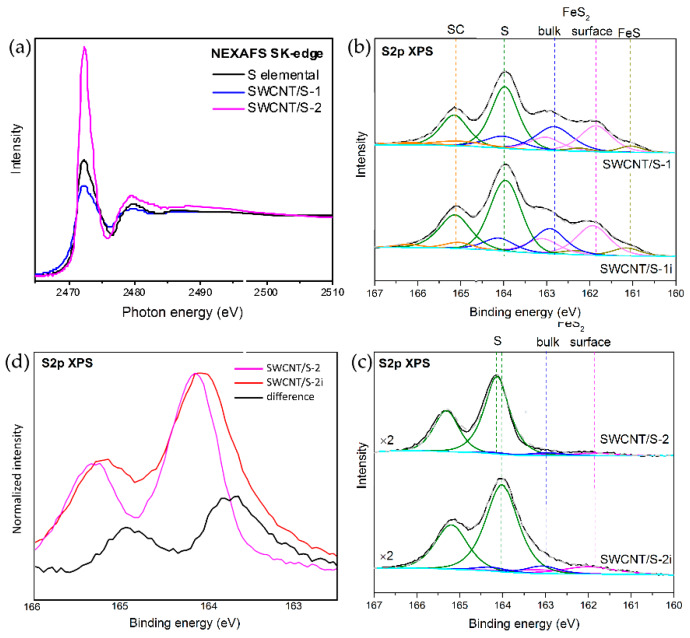
S *K*-edge NEXAFS spectra of SWCNT/S-1 and SWCNT/S-2 samples (**a**). S 2*p* XPS spectra of sulfur-contained SWCNTs before and after illumination (**b**) and (**c**). Normalized S 2*p* XPS spectra of for SWCNT/S-2 and SWCNT/S-2i samples and their difference (**d**).

To understand the electronic state of sulfur, the S *K*-edge NEXAFS and S 2*p* XPS spectra have been studied (Figure 5). The most apparent difference between S *K*-edges is the intensity of the white line (Figure 5a). As compared with the value for elemental sulfur, it slightly decreased in the SWCNT/S-1 spectrum and noticeably enhanced in the SWCNT/S-2 spectrum. Moreover, the sulfur absorption edge of SWCNT/S-1 and SWCNT/S-2 samples is shifted toward lower and higher values of photon energy with respect to elemental sulfur, respectively. Hence, the electronic states of external and encapsulated sulfur are different. In particular, the external sulfur acts as electron acceptor for SWCNTs, while the encapsulated sulfur donates electrons to nanotube. The absence of a visible shift of the XPS C 1*s* line for SWCNT/S-2 as compared to the initial SWCNTs could be attributed to a not enough high filling of the nanotubes.

**Figure 6:**
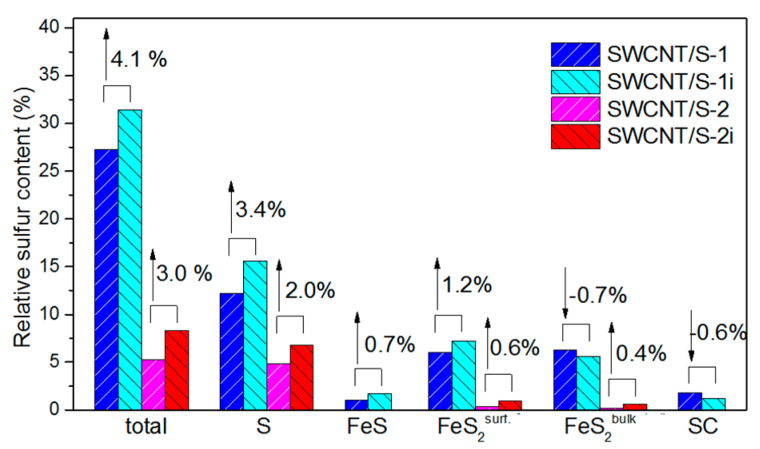
XPS sulfur content in SWCNT/S-1 and SWCNT/S-2 samples before and after irradiation. Numbers and arrows located above columns show increase (up arrows) or decrease (down arrows) of the sulfur content after irradiation.

The S 2*p* XPS spectra also showed the obvious difference in the chemical state of sulfur in SWCNT/S-1 and SWCNT/S-2 samples (Figure 5b,c). Deconvolution of the spectra by spin-orbit doublet with the 2*p*_3/2_ and 2*p*_1/2_ components allowed revealing different sulfur forms, which relative content is showed in Figure 6. In both cases, the major contribution comes from the doublet attributed to S-S bonds. Because the major state of sulfur in SWCNT/S-1 sample is bulk deposited particles, the binding energies of S doublet (XPS S 2*p*_3/2_ and 2*p*_1/2_) coincided with the values for elemental sulfur (*ca.* 164.0/165.2). When mainly the encapsulated sulfur is presented, S XPS lines were shifted by 0.2 eV to the higher energy with respect to their position in SWCNT/S-1 indicating the charge transfer from the encapsulant to the SWCNT wall that agrees with the NEXAFS data and the assumption made in earlier works [17,18,28]. The shoulder appeared at higher binding energy with respect to the main peaks represents sulfur atoms bounded with carbon (165.1/166.3 eV), which are absent in the SWCNT/S-2 sample. At the lower binding energies, the SWCNT/S-1 spectrum showed the lines from iron sulfides. Doublets at 162.8/164.0 and 161.8/163.0 eV are due to bulk and surface sulfur atoms in FeS_2_, respectively; doublet at 161.0/162.2 eV corresponds to FeS [46]. These doublets constituted about half of the total sulfur content of the SWCNT/S-1 sample (*ca.* 13.3%). In case of SWCNT/S-2 purified from iron using magnetic treatment, the content of sulfides did not exceed 0.5%.

### 3.2. Light Irradiation of Sulfur-Contained SWCNTs

Light irradiation of the SWCNT/S-1 and SWCNT/S-2 samples was carried out *in situ*. The C *K*-edge NEXAFS and XPS spectra were recorded from exactly the same areas, which have been used for the NEXAFS and XPS studies of the samples before the exposure. It guaranteed that the changes detected in the chemical states of carbon and sulfur after illumination is not due to the sample heterogeneity, but are caused by some transformations, which took place due to the treatment.

The NEXAFS C *K*-edge spectra of SWCNT/S-1i and SWCNT-S-2i samples exhibited enhancement of π* resonance and suppression of peaks *a* and *b* in comparison with that for SWCNT/S-1 and SWCNT/S-2, respectively (Figure 4). This means that the illumination treatment removed functional groups from SWCNTs. At the same time, the walls of nanotubes maintained their integrity, as evidenced by the sharpness of π* and σ* resonances and the C 1*s* line.

Our awaiting was that the continuous illumination of samples with high-brilliance non-monochromatized light should evaporate the sulfur decreasing its local content. However, the overall XPS showed increase of the relative sulfur concentration by 4.1% and 3.0% for SWCNT/S-1i and SWCNT/S-2i samples, respectively, compared to the values before the treatment (Figure 3 and Figure 6). The S 2*p* spectra (Figure 5b,c) showed that this increment arose mainly from the growth of pure sulfur content. Moreover, the binding energy of S-S line in SWCNT/S-2i sample slightly decreased with respect to the value for SWCNT/S-2 sample. This effect is more clear from the difference between the normalized spectra of SWCNT/S-2i and SWCNT/S-2 samples plotted in Figure 5d. The appearance of notable components with the lower binding energy than that in the spectrum of the sample before illumination indicated that the encapsulated sulfur, which is the majority, extracted from the SWCNT cavity to the surface of nanotubes. The content of sulfides also increased by *ca.* 1% for both samples, while the content of sulfur atoms bounded with carbon in SWCNT/S-1 sample slightly decreased. Taking into account surface sensitivity of the XPS spectra we believe that sulfur moved from the bundles and cavities of nanotubes to the outer surface.

The effective absorption of visible and near-infrared light makes CNT perspective as a heating agent. In particular, laser irradiation on CNTs ablated cancer cells [47,48,49] and provoked significant rise of local temperature of tissues containing injected nanotubes [50,51,52] and myosin molecules adsorbed above them [53]. The laser irradiation induces very fast, high temperature thermal cycles in CNT, which even could produce nanodiamonds [54]. Other study showed that the flashing with several high-power pulses for 29 ns (pulse energy density 790 mJ/cm^2^, pulse width 6 ns) heated the surface temperature of CNT film up to *ca.* 800 °C [55]. The treatment with the continuous infrared laser was shown to heat CNT network to over 170 °C in less than 2 s [56]. We have illuminated the samples by non-monochromatized synchrotron radiation light, which contains photons of different energies with the total photon flux of order of 10^14^ photons/s. From visible to ultraviolet ranges, the photon flux varies in the interval 2 × 10^12^–5 × 10^13^ photons/s. Since the beam size is ca. 1 × 1 mm^2^, the total light intensity in the range of 1–10 eV can be estimated as 50 mJ/cm^2^ that is one order of magnitude smaller than the previously reported laser fluence required for melting of CNTs (*ca.* 400 mJ/cm^2^) [54]. However, we did not see any significant impact of the synchrotron irradiation on the carbon network, while the surface content and chemical states of sulfur changed (Figure 3 and Figure 5). Taking into account that orthorhombic sulfur melts at a rather low temperature (*ca.* 115 °C) than nanotubes, and understanding that the heating of sample occurred not only due to the CNT absorption mechanism but also as a result of knocking-on of sulfur atoms by high-energy photons, the melting of sulfur in SWCNT/S-1i and SWCNT/S-2i could be proposed. Due to the pressure difference, melted species moved from the bundles and cavities of nanotubes to the outer surface increasing its content as XPS showed. At that, the content of S-S-component in S 2*p* XPS spectra increased by 28% and 42% for SWCNT/S-1i and SWCNT/S-2i samples with respect to the value before the irradiation. This indicates the higher effect of irradiation on sulfur encapsulated inside the cavities of SWCNTs that agrees with the stronger heating of nanotubes interior during laser heating predicted theoretically [57] and lower stability of sulfur encapsulated into outer SWCNTs of a bundle relative to the surface-deposited sulfur demonstrated by the TG data. Moreover, one can expect that low-power laser irradiation also could cause the exclusion of sulfur or another light element from the cavities of SWCNTs.

## 4. Conclusion and Prospects

Materials containing sulfur and SWCNTs have been obtained by the ampoule synthesis. TEM, Raman, TG and X-ray data showed that sulfur effectively encapsulated inside SWCNTs, while, in the presence of residual catalyst particles, a significant proportion of sulfur is consumed on the formation of iron sulfides. Our results distinctly showed the charge transfer from encapsulated sulfur to walls of SWCNTs. The illumination of sulfur-contained SWCNTs with zero-order light from the Russian-German dipole Beamline of BESSY II, HZB for 30 s enhanced the surface sulfur content by 3–4%. The comparison of XPS S 2*p* spectra of the samples before and after the treatment showed the transformation of encapsulated sulfur into external sulfur and the increase in the concentration of sulfides. These effects were related to sulfur transport outward of SWCNTs bundles due to the light-induced heating of nanotubes followed by sulfur melting. Our results definitely showed that encapsulated species could be excluded from the cavity of SWCNT at rather moderate heating. This observation can be useful for hyperthermic applications of nanotubes and target drug delivery systems.
